# Seven candidate interventions to address abuse of older people

**DOI:** 10.1093/ageing/afaf248

**Published:** 2025-09-08

**Authors:** Laura Campo-Tena, Jeffrey H Herbst, Wan Y Choo, David Burnes, Mélanie Couture, Fatemeh Estebsari, Christelle S L Kafando, George Rouamba, Marie-Madeleine Simbreni, Elsie Yan, Yongjie Yon, Christopher Mikton

**Affiliations:** Institute of Criminology, Cambridge, University of Cambridge, UK; Centers for Disease Control and Prevention, Atlanta, GA, USA; Department of Social and Preventive Medicine, Universiti Malaya, Kuala Lumpur, Federal Territory of Kuala Lumpur, Malaysia; Factor-Inwentash Faculty of Social Work, University of Toronto, Toronto, Ontario, Canada; École de Travail Social, Université de Sherbrooke, Sherbrooke, Quebec, Canada; Department of Medical Surgical Nursing, School of Nursing and Midwifery, Shahid Beheshti University of Medical Sciences, Tehran, Iran; Institut Supérieur des Sciences de la Population, Université Joseph Ki-Zerbo, Ouagadougou, Centre Region, Burkina Faso; Department of Sociology, Universite Joseph Ki-Zerbo, Ouagadougou, Centre Region, Burkina Faso; Department of Sociology, Universite Joseph Ki-Zerbo, Ouagadougou, Centre Region, Burkina Faso; Department of Applied Social Sciences Hong Kong, The Hong Kong Polytechnic University, Hong Kong, China; Division of Country Health Policies and Systems, World Health Organization Regional Office for Europe, Copenhagen, Capital Region of Denmark, Denmark; Department of Social Determinants of Health, Division of Healthier Populations, World Health Organization, Geneva, Switzerland

**Keywords:** abuse of older people, intervention accelerator, systematic screening and assessment, evaluability assessment, global, older people

## Abstract

The Abuse of Older People – Intervention Accelerator (AOP-IA) project aims to accelerate the development of effective interventions to prevent and reduce AOP aged 60 and older within the framework of the United Nations Decade of Healthy Ageing (2021–2030). The AOP-IA was launched in response to the global need for interventions with proven effectiveness, as few existing approaches have been rigorously evaluated. This paper focuses on the first two phases of the AOP-IA project, which involved conducting a systematic search, screening and evaluation process to identify candidate interventions ready to be rigorously evaluated in future stages of the project, as well as establishing a network of intervention developers. The identification of interventions included an initial screening of 13 926 records and two rounds of evaluations by an expert panel. From this process, 89 promising interventions were identified, and subsequently, seven candidate interventions were selected for more rigorous scientific testing and evaluation. An adapted version of the Systematic Screening and Assessment Method was used to identify these interventions. The AOP-IA project demonstrates that interventions to prevent and reduce abuse of older adults exist in a variety of settings and countries, and that several interventions are ready for a rigorous evaluation to support continual programme improvement by intervention developers and long-term sustainability and scale-up globally.

## Key Points

- The AOP-IA was launched in response to the global need for interventions with proven effectiveness.
- Interventions to prevent abuse of older adults exist in a variety of settings and countries.
- Seven candidate interventions were selected for more rigorous scientific testing and evaluation.

## Introduction

Abuse of older people (AOP) is defined as any single or repeated act—or lack of appropriate action—occurring within a relationship of trust that causes harm or distress to an older person [1]. According to the World Health Organisation (WHO), one in six individuals aged 60 and older in the community experiences some form of abuse or neglect each year [2]. The issue is even more severe in institutional settings, with two-thirds of staff in facilities like nursing homes admit to committing abuse [3]. With the global population ageing rapidly, the number of older adults at risk is set to increase significantly unless effective preventive measures are implemented.

In response to this urgent issue, WHO initiated in 2022 a priority setting exercise convening a group of 50 international experts and stakeholders who unanimously recognised the development of effective interventions as a top priority for preventing AOP globally [4]. Previous research also highlighted that there is a lack interventions with demonstrated effectiveness in high-quality evaluations [5–11].

### The abuse of older people intervention accelerator project

Responding to the urgent global need for effective interventions to prevent AOP, the WHO launched an initiative to establish an ‘intervention accelerator’ as part of the United Nations Decade of Healthy Ageing (2021–2030). The main aim of this project is to accelerate the development of effective interventions to prevent and reduce AOP in various settings across countries of different income levels [4]. The Abuse of Older People – Intervention Accelerator (AOP-IA) is a consortium of experts in preventing and responding to AOP in diverse regions of the world.

The intervention accelerator is made up of four phases, each with specific objectives. Phase 1 aims to identify and create a database of promising intervention approaches. From this database, a subset of candidate interventions is selected for an evaluability assessment to identify the most promising interventions. Phase 2 focuses on creating and maintaining a network of developers, implementers and evaluators of these intervention approaches. In Phase 3, the selected candidate interventions are refined and adapted, then evaluated through rigorous testing. Finally, Phase 4 involves creating and maintaining an online, living ‘portfolio’ of interventions.

The current paper describes Phases 1 and 2 of the intervention accelerator process, including a description of processes to identify candidate interventions, results of evaluability assessments of candidate interventions and then the development of a list of promising interventions that are ready for more rigorous testing and evaluation (cf. [12, 13].

## Method

### The systematic screening and assessment method

The Systematic Screening and Assessment (SSA) Method [12] provides an understanding of the potential impact, feasibility and readiness for evaluation of interventions, and ensures that resources are directed towards rigorous testing of the most promising interventions to expand the evidence base [13].

**Figure 1:**
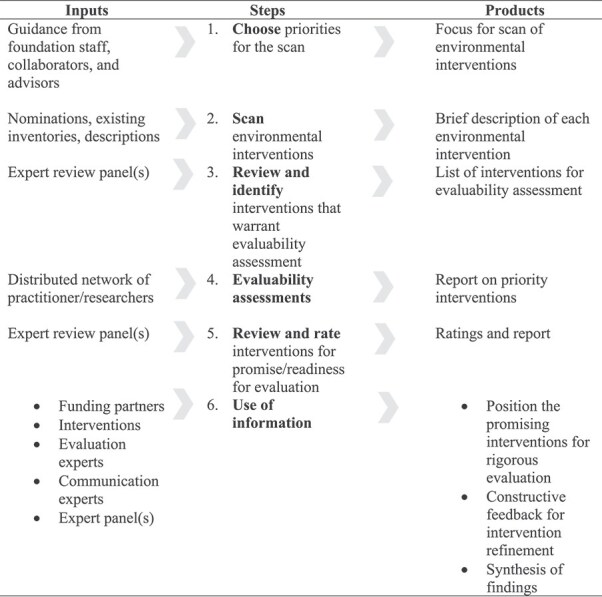
The SSA Method according to Leviton & Gutman [12].

The general SSA Method has six main steps as displayed in Figure 1. Application of the SSA Method to the findings of a recent literature search on AOP prevention [14] is expected to yield candidate interventions ready for additional evaluation and pilot testing.

### Adaptation of the SSA method: Steps and deviations

**Figure 2:**
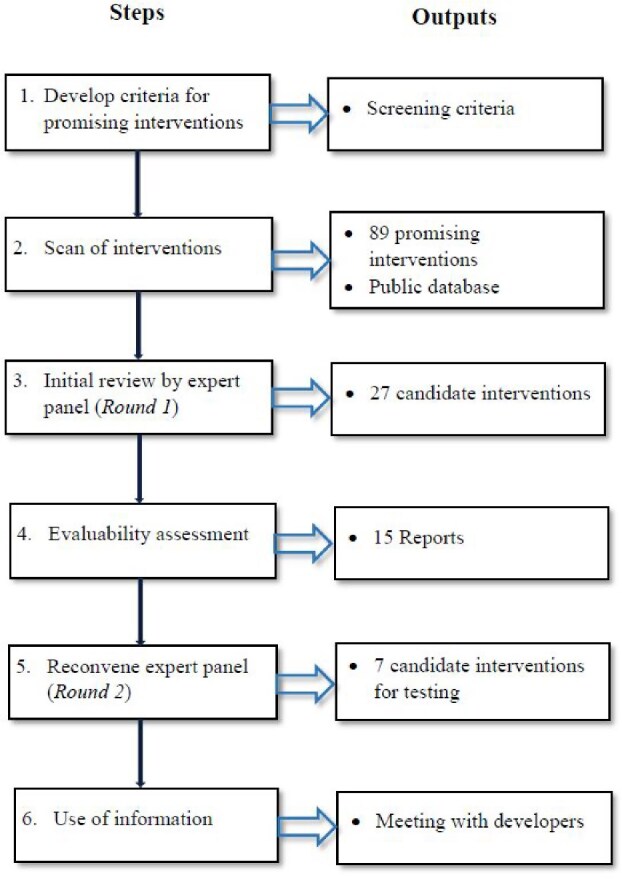
Adapted Systematic Review and Assessment Method for the WHO’s AOP-IA.

An adapted version of the SSA Method (see Figure 2), informed by a prior application in violence prevention, was used to identify interventions that are candidates for more rigorous scientific testing and evaluation [15].

The AOP-IA developed a protocol detailing the adapted SSA Method. Campo-Tena and colleagues [14] describe Steps 1 and 2. The current paper specially focuses on Steps 3 to 6, as described above.

Whilst we aimed to adhere closely to the recommended SSA methods, we had to make adjustments based on available resources. Time constraints were a significant factor; the original SSA Method is intended to span multiple years, but we had ⁓16 months to complete all six steps. Funding limitations also influenced our approach. Leviton and Gutman [13] suggest that a comprehensive study of intervention effectiveness requires a budget of at least $2 million USD, whereas we operated with a fraction of this amount plus *pro bono* support from a cadre of researchers, including support from graduate students and interns. Consequently, some steps were less exhaustive than originally planned. For example, we were unable to develop the logic models (Step 4 of the original SSA Method) and plan to do this when the candidate interventions undergo more rigorous testing, in collaboration with the teams that developed them.

The timing of some steps differed from the original SSA Method. Specifically, instead of assessing sustainability and organisational capacity in Step 3, as in the original method, these were evaluated later in Step 5, after collecting more information from documents and interviews.

### Ethics

The WHO Ethics Review Committee reviewed this project and determined that the submission of an ethics application was not necessary.

### Risk of bias

To mitigate the risk of bias during the selection process of candidate interventions, we randomly assigned interventions to collaborators for evaluation. If any collaborator had a perceived conflict of interest, they were excluded from evaluating the intervention at all stages of the SSA process to ensure impartiality.

### Procedure

First, we developed the selection and screening criteria for identifying promising interventions based on the agreed criteria (Supplementary Material—Appendix S1). Second, we conducted searches using a four-step strategy, as detailed in Campo-Tena *et al.* [14]. Since no interventions were identified in sub-Saharan Africa, our collaborators in Burkina Faso conducted a more in-depth review with an adjusted search strategy.

Next, an evaluability assessment was conducted of all promising interventions identified that met inclusion criteria as specified in Supplementary Material—Appendix S2. For this, an expert panel formed by collaborators from the AOP-IA consortium was actively involved in applying the criteria in two rounds of evaluations. The evaluability assessments were based on a review of documents and online interviews with the original intervention developers. The criteria included feasibility of implementation, feasibility of adoption, transferability, sustainability of the strategy, ethical approach, reach to the target population, population plausibility, potential impact, sustainability of the intended outcome(s) and evaluation potential. Finally, we reconvened the expert panel to review the evaluability assessment findings, and select the final candidate interventions for further testing and evaluation in subsequent years of the project (i.e. Phase 3 of the AOP-IA).

## Methodological considerations for evaluability assessment

This step included the collection of documents on the intervention and online interviews a with the intervention developers.

### Documentation process

Materials for the evaluability assessment covered a detailed description of the intervention, a well-specified logic model or programme theory, identification of main stakeholders involved in intervention and their expectations, and a description of activities involved in intervention implementation (including timelines), a description of available materials (e.g. protocols, training materials, participant handouts, etc.), an estimate of all resources required (i.e. human, institution/infrastructure, equipment, financial, etc.) and full details of any previous evaluations. This information was mainly identified through online web searches by the AOP-IA Consortium, with unavailable documents requested directly from the intervention developers by email.

To store and organise the data gathered in the evaluability assessment, a coding sheet following the TIDieR (Template for Intervention Description and Replication) checklist [16], was used. Secure folders were used in a WHO-hosted shared drive to store all documents.

### Interviews

Interviews with the selected interventions’ developers were conducted online, which last ⁓1 h. They were based on an adaptation of a semi-structured SSA guide developed by the U.S. Centres for Disease Control and Prevention (CDC) for hospital-based youth violence prevention interventions [15] (Supplementary Material—Appendix S3).

### Reports

A short internal report was produced for each intervention based on the information obtained from the evaluability assessment (i.e. documentation process and interviews) using a report template (Supplementary Material—Appendix S4). The reports covered the following topics: feasibility of implementation and adoption; transferability; sustainability of the strategy; ethical approach; reach to the target population; population plausibility; potential impact; sustainability of the intended outcome(s) and evaluation potential. The report content mirrored the criteria for Round 2 (i.e. Supplementary Material—Appendix S2).

### Round 2 evaluation

The goal of this step was to identify the most promising ‘candidate interventions’ to be refined, adapted and evaluated in subsequent years of the project.

Each candidate intervention was assessed by two independent members of the AOP-IA Consortium using a scoring system developed by the AOP-IA Consortium (Supplementary Material—Appendix S5). A provisional ranking of interventions resulted from this process. Results were discussed with the AOP-IA Consortium, and a portfolio of seven interventions was selected for the next phase of the project. Similar to Round 1 evaluation, aspects considered for the ranking, and final selection, of interventions during Round 2 included: (1) High evaluation scores and agreement between experts; (2) Geographical origin, income level and adaptability to other settings; (3) Diversity in abuse type, target population, interventions deliverers and delivery mode and (4) A preference for community over institutional settings, given that only 5% or less of older adults live in institutions [17].

## Results

### Adapted SSA step 1: Develop criteria for promising interventions

The four-step search strategy developed for the AOP-IA produced 13 926 records.

### Adapted SSA steps 2 and 3: Scan of interventions and initial review by expert panel (round 1)

**Figure 3:**
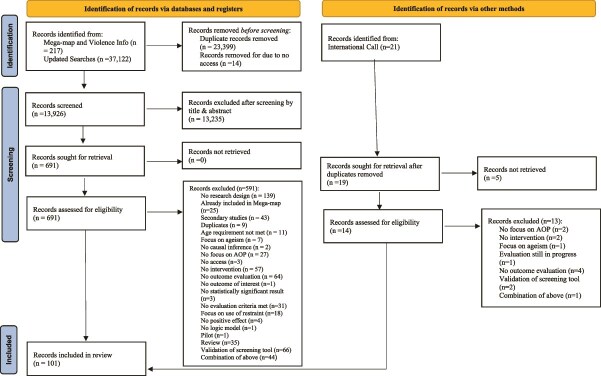
Selection Process Diagram—Mega-map and Violence Info, Updated Searches and International Call, WHO AOP-IA.

The 13 926 records were screened by two independent reviewers. A total of 89 promising interventions reported in 101 unique publications were obtained through this process (see Figure 3 and included in a database of promising interventions to prevent and respond to AOP (see: https://explore.decadeofhealthyageing.org/aop-ia). Additional details are reported in Campo-Tena *et al.* [14].

### Adapted SSA step 4: Evaluability assessment

We scored the 89 promising interventions on several dimensions as described above, ranked them, and then contacted the developers of the interventions based on a combination of considerations to ensure diversity (ranking, geographic diversity, type of abuse addressed, etc.). This resulted into 27 developers (30% of 89) candidate interventions designed to prevent or reduce abuse amongst older adults eventually contacted. This also included the developers of an intervention in Burkina Faso, the sole intervention identified in an additional review of interventions in sub-Saharan Africa conducted for this project. Of the 27 intervention developers, 14 (56%) were interviewed.

Ten developers did not respond to our initial email and two follow-up email reminders. One developer declined to participate, and another was unavailable for an interview within our timeline. One developer responded and provided all the required documentation for the Round 2 evaluation, but no interview was conducted.

### Adapted SSA step 5: Reconvened expert panel and review of evaluability assessments (round 2)

**Figure 4:**
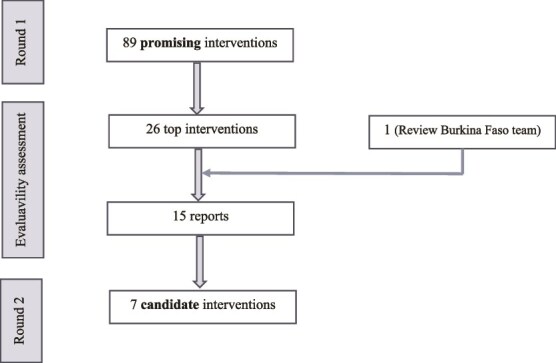
Diagram of Intervention Selection, WHO AOP-IA.

The AOP-IA Consortium was reconvened to review the reports and rate each intervention (see Supplemental Material—Appendix S2). During this step, 15 interventions (14 of which included interviews) were assessed and thoroughly discussed. Amongst these, seven candidate interventions were ultimately selected for Phase 2 of the AOP-IA project (See Figure 4).

### Characteristics of the candidate interventions

Table 1 provides an overview of the main characteristics of the selected interventions.

(1) RISE (Canada and USA): A community-based intervention using restorative approaches, motivational interviewing, supported decision-making and teaming (strengthening social supports) to support older adults at risk of or experiencing abuse.
(2) PEOPLE (Iran): A group-based, digitally enhanced programme that empowers older adults to prevent abuse through social support and health-promoting behaviours.
(3) SAFE (USA): An individual intervention addressing financial abuse through community education, recovery support and personalised financial coaching.
(4) PROTECT (USA): A therapeutic intervention using problem-solving and anxiety management to reduce depression in older adults who have experienced abuse.
(5) PEC-Sakoula (Burkina Faso): A community programme supporting older victims of witchcraft accusations through basic care, literacy and income-generating activities.
(6) SEARCH (USA): A training-based intervention for nursing home staff to prevent and respond to resident-to-resident violence in long-term care.
(7) I-NEED (Malaysia): A blended training programme, combining face-to-face sessions with a digital component, designed to equips primary care nurses in institutional settings to identify and respond to abuse of older adults.

**Table 1:** Main characteristics of seven selected candidate interventions by WHO AOP-IA

Short name	Associated references	Community vs Institutional	Round 1 ranking position (out of 89)	Round 2 ranking position (out of 15)	Type(s) of violence	Level of Intervention^a^	Country	WHO Region	Country Income Level	Target group	Delivered by which group	Mode of delivery
RISE	Burnes *et al*. (2022) [18]	Community	17	2	Any	Secondary	United States, Canada	Americas	High income	Victims, alleged harmers and victim-harmer relationship	RISE advocates	In-person and/or telephone/virtual-based modes
PEOPLE	Estebsari *et al*. (2018) [19]	Community	4	10	Any	Primary	Iran (Islamic Republic of)	Eastern Mediterr-anean	Lower middle income	Potential victims of AOP	Other healthcare workers	Face-to-face, Group, Digital
SAFE	Hall *et al*., (2022) [20]	Community	26	11	Financial, including scams and fraud	Primary/Tertiary	United States	Americas	High income	Potential victims of AOP	Staff and the research team	Face-to-face, Individual
PEC-Sakoula	Fonds commun genre (2021) [21]	Community	NA	12	Accusations of witchcraft	Tertiary	Burkina Faso	African	Low income	Victims of AOP	Nurses, social worker, farm technician, garden specialist, trainer	Face-to-face, Individual
PROTECT	Sirey *et al*. (2015a) [22]; Sirey *et al*. (2015b) [23]; Sirey et al. (2021) [24]	Community	5	14	Any	Tertiary	United States	Americas	High income	Victims of AOP	Social workers	Individual, Face-to-face
SEARCH	Teresi *et al.*, (2013) [25]	Institutional	7	1	Resident-to-resident in long-term care institutions)	Secondary	United States	Americas	High income	Professional caregivers	Other healthcare workers	Group, Face-to-face
I-NEED	Mydin *et al*. (2022) [26]	Institutional	1	9	Any	Secondary	Malaysia	Western Pacific Region	Upper-middle income	Professional caregivers	Nurses, physicians, other healthcare workers	Face-to-face, Digital

^a^Primary interventions aim to prevent abuse before it occurs; secondary interventions target early signs or risks to stop abuse from escalating; tertiary interventions address the consequences of abuse and seek to prevent its recurrence.

Abbreviations. RISE: Relational, Individual, Social and Environmental levels of ecological influence; PEOPLE: Prevention through Empowerment for Protecting Older People’s Lives and Engagement; SAFE: The successful ageing through financial empowerment (SAFE) programme; PROTECT: Providing Options to Elderly Clients Together; SEARCH: Support, Evaluate, Act, Report, Care plan and Help; I-NEED: Improving Nurses’ dEtection and managEment of elDer abuse and neglect; WHO: World Health Organization; AOP: Abuse of Older People; APS: Adult Protective Services.

The candidate interventions represent a diverse range of approaches to addressing AOP across different settings, violence types and target groups.

### Settings

The majority of interventions (n = 5) are community-based. The two remaining interventions, SEARCH and I-NEED, are institutional-based, focusing on preventing and responding to abuse in long-term care settings.

### Types of violence

Most interventions address any form AOP, including RISE, PEOPLE, PROTECT and I-NEED. SAFE specifically addresses financial abuse, including scams and fraud, whilst PEC-Sakoula focuses on accusations of witchcraft, which is a common form of AOP in some regions in the sub-Saharan Africa. SEARCH targets resident-to-resident (Although resident-to-resident aggression may not align with all definitions of abuse of older people—particularly those requiring an expectation of trust—it was included due to its growing recognition as a significant form of harm in institutional settings and the broader duty of care inherent to these environments.) violence in long-term care institutions.

### Levels of intervention

The interventions span primary, secondary and tertiary prevention levels. PEOPLE and SAFE are primarily focused on prevention, whilst RISE, SEARCH and I-NEED focus on secondary prevention. Tertiary interventions, such as PEC-Sakoula and PROTECT, are aimed at addressing existing cases of abuse. SAFE integrates both primary and tertiary approaches.

#### Geographic representation

The selected interventions come from various regions, with four based in the United States (SAFE, PROTECT, RISE and SEARCH) and Canada (RISE). PEOPLE is based in Iran, and I-NEED is based in Malaysia. Last, there is one intervention based in Burkina Faso. These interventions cover diverse WHO regions, including the Americas, Eastern Mediterranean, African and Western Pacific regions.

#### Target groups

The interventions target different groups affected by or at risk of abuse. PEOPLE, and SAFE focus on any older adults that are not necessarily victims but at risk of being victims of abuse, whilst PEC-Sakoula and PROTECT specifically address victims of AOP. RISE works with older adults who are either at risk of, or have experienced, abuse. SEARCH targets nursing home staff to prevent resident-to-resident abuse, and I-NEED focuses on institutional staff, specifically primary care nurses.

#### Delivery methods

Most interventions are delivered face-to-face, either individually or in group settings. PEOPLE and I-NEED also incorporate digital delivery methods, adding flexibility and accessibility. SAFE, PEC-Sakoula and PROTECT are primarily delivered in individual settings, whilst PEOPLE and SEARCH use group-based approaches. RISE is delivered using in-person and/or telephone/virtual-based modes.

### Adapted SSA step 6: Use of information including dissemination and plans for future rigorous evaluation

The developers of the seven selected candidate interventions were invited to be part of a newly created international network of developers, implementers and evaluators committed to further refining, adapting and testing candidate interventions approaches to prevent or reduce abuse against older adults.

### Feasibility and sustainability

Feasibility of implementing the seven interventions in different countries and cultural contexts varies. Interventions like RISE, SEARCH and I-NEED exhibit higher feasibility due to their flexible, straightforward designs, manageable training demands and adaptability across settings. RISE’s flexibility and minimal need for highly specialised staff facilitate its transferability, whilst SEARCH’s clear training modules and I-NEED’s brief, nurse-friendly format support easier adoption. In contrast, programmes such as PEOPLE and PEC-SAKOULA encounter more significant cultural and systemic challenges; PEOPLE must navigate political and cultural resistance, especially related to norms around older adults, whereas PEC-SAKOULA requires complex multi-partner mobilisation to address rooted beliefs like witchcraft, complicating replication. SAFE’s intensive resource needs and reliance on community partnerships make it feasible only where robust infrastructure exists, whilst PROTECT balances simplicity and adaptability but may face variable cultural openness to mental health topics. Across all interventions, successful implementation depends on how well their complexity, resource demands and cultural fit align with the target setting’s capacities and constraints.

Sustainability reflects similar themes, hinging on stable funding, integration into existing systems and the ability to maintain operations without extraordinary leadership or resources. RISE has benefitted from steady state funding, aiding long-term viability, whilst PEOPLE’s sustainability depends on continued institutional and financial backing beyond initial self-funded efforts. SAFE faces ongoing challenges due to its resource-intensive nature despite national recognition and virtual adaptations enhancing its prospects. PROTECT’s simple training and flexible delivery contribute to its resilience, especially during disruptions like the COVID-19 pandemic. PEC-SAKOULA’s sustainability relies on the centre’s ability to self-finance through marketable production. SEARCH appears sustainable given the persistent need to address behavioural issues in nursing homes and its modular, updateable training. I-NEED shows promise through its integration into existing healthcare structures and efficient format, though cost-effectiveness and initial reliance on support from the public healthcare system remain considerations. Overall, sustainability is strongest where interventions combine reliable funding, system integration and adaptable models, whereas those demanding high resources or complex leadership face greater sustainability challenges.

## Discussion

The WHO’s AOP-IA Project addresses one of five key priorities outlined in Tackling AOP: Five Priorities for the United Nations Decade of Healthy Ageing (2021–2030)—developing and scaling cost-effective solutions. Through an intervention accelerator and the SSA methodology, the project aims to identify interventions with strong potential to prevent or reduce AOP.

Despite previous reviews noting a lack of rigorously evaluated interventions [5–8, 10, 11, 27], our process, using databases, environmental scans, expert panels and developer interviews, found growing evidence of evaluated programmes. Several are now ready for refinement, adaptation and further rigorous evaluation of effectiveness.

This paper outlines our adapted SSA process. In Round 1 (Steps 1–3), we identified 89 promising interventions, published in a public database. Round 2 (Steps 4–5) involved detailed evaluability assessments of 14 interventions, plus one from Burkina Faso, resulting in the selection of seven interventions for potential further testing, depending on available resources.

The adapted SSA methodology, supported by expert review, helps build an evidence base for AOP prevention. Time and funding constraints required us to adapt SSA steps, but expert consultation and *pro bono* support helped preserve methodological rigour. This work should be seen as a foundational prioritisation effort, with future stages planned to validate and improve the approach. The seven interventions were selected based on feasibility, alignment with project goals and readiness for scale, though this may affect generalizability and introduce some selection bias.

The selected interventions vary by setting, violence type and target population. Most are community-based, reflecting the fact that older adults typically live outside institutions. They span all prevention levels (primary, secondary and tertiary) and employ various delivery models, making them adaptable but also posing implementation challenges.

Each of the seven interventions offers promise but also presents unique challenges. For example, RISE, SEARCH and I-NEED are notable for ease of training and adaptability, whilst PEOPLE, SAFE and PEC-SAKOULA may face challenges with cultural fit, resource demands and leadership requirements. Local stakeholder engagement, cultural sensitivity and integration into existing systems may be essential for success, especially in resource-limited contexts. Virtual delivery models, as seen in PROTECT and SAFE, may help increase reach. Future work could prioritise scalable, low-cost interventions supported by robust monitoring, leadership development and stakeholder ownership.

The project faced several limitations. First, initial searches relied on peer-reviewed literature and web-based sources, though expanded searches and a global call for submissions broadened scope. Still, four of the seven selected interventions were based in the United States, likely reflecting the country’s sustained funding for AOP prevention. However, promising interventions were also identified in Asia and Africa.

Second, some SSA steps were abbreviated due to resource constraints; these will be integrated into future project phases. Third, no peer-reviewed evaluations by NGOs were identified, possibly due to limited evaluation capacity or publishing challenges.

The strength of the AOP-IA project is its pragmatic approach. By adapting SSA to time and budget constraints, we preserved research quality and demonstrated that impactful work is feasible even in limited-resource settings. This flexible framework offers a scalable model for future initiatives.

In summary, the AOP-IA Project confirms the existence of promising interventions across various global contexts, many of which are ready for more rigorous testing to support programme improvement, sustainability and broader impact.

## Supplementary Material

aa_25_0748_File006_afaf248
