# Supplementary material for: Seven candidate interventions to address abuse of older people
Source: Age Ageing. 2025 Sep 8;54(9):afaf248. doi: 10.1093/ageing/afaf248 (PMC12416808; doi:10.1093/ageing/afaf248)
Supplement: aa_25_0748_File006_afaf248 [file aa_25_0748_file006_afaf248.docx]

**Supplementary Material**

**Seven Candidate Interventions to Address Abuse of Older People**

**Table of Contents**

[Appendix 1. Criteria for Screening of Promising Interventions 2](#_Toc202948742)

[Appendix 2. Criteria for Round 2 8](#_Toc202948743)

[Appendix 3. Interview guide 9](#_Toc202948744)

[Appendix 4. Report Template 13](#_Toc202948745)

[Appendix 5. SSA Step 5 (Round 2) – Scoring System 15](#_Toc202948746)

### Appendix 1. Criteria for Screening of Promising Interventions

|  | Inclusion | Exclusion | Definitions/explanation |
| --- | --- | --- | --- |
| Type of study | - Primary study consisting of an outcome. evaluation of an intervention to prevent or respond to abuse of older people. | - Reviews of multiple studies of any sort (e.g., systematic review, narrative review, meta-analysis, etc.) & protocols. |  |
| Beneficiaries of Intervention | - Victims of abuse of older people (i.e., people 60 years and over). - If the population of intervention beneficiaries includes victims of abuse of older people (60+) or of intimate partner violence (who are 60+), but also includes other age groups, the relevant findings must be available in a disaggregated form for people 60+ (i.e., findings for people 60+ must be presented separately). - If the age cut-off is less than 60 (e.g., 55) but 50 or more and the mean age of the sample is over 65, then the study should be included, even if findings are not disaggregated for those 60+. | - Any population group that does not meet the inclusion criteria, i.e.,   - Population includes people under the age of 60 and findings for those 60+ are not disaggregated; AND   - If age cut-off <60 but 50 or more, mean age is 65 or less. | Abuse of older people:   - Definition of abuse of older people/elder abuse: a single or repeated act or lack of appropriate action, occurring within any relationship **where there is an expectation of trust** which causes harm or distress to an older person (i.e., 60+). The main forms of elder abuse generally recognized, and which can occur in the community and institutional settings are physical, psychological, financial/material, sexual abuse and neglect, systemic/organizational abuse, and poly-victimization. - This definition of abuse of older people includes intimate partner violence perpetrated against a person 60 years or older. |
| Intervention Participants | - Populations which the interventions to prevent or respond to the **abuse of older people** are aimed at including: - Perpetrators of abuse of older people (of any age, e.g., spouse/intimate partner, family, neighbours, non-professional and professional caregivers). - Victims of abuse of older people (i.e., people 60 years and over). - “Concerned others”, i.e., individuals who are involved in the situation but have not perpetrated the abuse, such as friends, neighbours, and relatives of the victim of abuse of older people (of any age). - Non-professional caregivers at risk of perpetrating abuse of older people (of any age, e.g., spouses/intimate partners, family, friends, neighbours, volunteers, etc.). - Professional caregivers at risk of perpetrating abuse of older people (of any age, e.g., staff in long-term care institutions, agency caregivers, nurses, social workers, physicians, administrators or managers for organisational/institutional abuse, etc.). - Non-caregivers at risk of perpetrating abuse of older people (of any age, e.g., spouse/intimate partner, family, friends, acquaintances, neighbours, etc.). - General population (of any age, e.g., awareness campaigns, community level interventions, system level interventions such as laws and policies). - Institutions themselves (rules and regulations governing care home, long term care facilities, hospitals, banks, etc.). | - Any population group perpetrating violence, crime or abuse against a person 60+ where there is no relationships in which there is an expectation of trust. This means, for instance, that interventions aiming to reduce older people’s victimization in street crime perpetrated by strangers, etc. are excluded. | Intervention participants:   - Individuals or groups who are directly involved in the intervention program or study. These participants are selected based on specific criteria relevant to the intervention's goals and objectives. The intervention participants can vary depending on the nature of the intervention and its target population. In the context of interventions to prevent or respond to the abuse of older people, the intervention participants can encompass a broad range of individuals, including perpetrators, older people, formal and informal caregivers, the general population, and the institutions themselves (see inclusion criteria in this row). |
| Type of abuse | - Physical. - Psychological (verbal/emotional), including accusations of witchcraft. - Intimate partner violence, including psychological and physical abuse. - Sexual (by any perpetrator). - Financial, including scams and fraud. - Neglect (including abandonment). - Systemic/organizational/or institutional abuse. - Resident-to-resident abuse. | - Self-neglect. - Ageism (e.g., stereotypes, prejudice, and discrimination based on age). - The use of restraints (physical & chemical) and seclusion. | - If an intervention aims both to prevent or respond to abuse of older people and other forms of violence (e.g., intimate partner violence in younger age groups) it will be included, provided findings are disaggregated for those 60+ (when relevant). - We will consider that financial/material abuse includes scams and frauds in as much as the older person has an expectation of trust – even if misplaced – in relation to the person the scammer or fraudster is purporting to be. |
| Intervention | - **All interventions** (i.e., primary, secondary, or tertiary prevention; or universal, selective/ed, and indicated) aiming to prevent or respond to **abuse of older people.** | Interventions that exclusively aim to address one or a combination of the following:   - Validation studies of tools to screen/identify/detect abuse of older people. - Ageism (e.g., stereotypes, prejudice, and discrimination based on age). | Intervention and intervention types   - The definition of i**ntervention** we will use will follow that of a “health intervention” which refers to an act performed for, with or on behalf of a person or a population whose purpose is to assess, improve, maintain, promote or modify health, functioning or health conditions. Types of interventions vary widely and range from narrowly focused interventions such as one-to-one psychological treatments to much broader interventions such as public health laws and policies. - **Primary, secondary, and tertiary prevention** interventions will be included: - Primary prevention – approaches that aim to prevent abuse before it occurs. - Secondary prevention – approaches that focus on the more immediate responses to abuse, such as pre-hospital care, emergency services or treatment for sexually transmitted diseases following a rape. - Tertiary prevention – approaches that focus on long-term care in the wake of abuse, such as rehabilitation of perpetrators and attempts to lessen trauma or reduce the long-term harms and disability associated with abuse in victims. - **Universal, selective, and indicated interventions** will be included: - Universal interventions – approaches aimed at groups or the general population without regard to individual risk; examples include media awareness campaigns delivered to the whole population. - Selected interventions – approaches aimed at those considered at heightened risk for abuse (having one or more risk factors for abuse); an example of such an intervention is training for staff in long-term care institutions about abuse of older people. - Indicated interventions – approaches aimed at those who have already demonstrated abusive behavior, such as treatment for perpetrators of abuse, to prevent recurrence; or those who have been victims of abuse to mitigate impact of abuse. |
| Setting | - All settings, including:   - Community.   - Institutions (e.g., long-term care institutions, hospitals, prisons or justice, etc.).   - Any others. |  |  |
| Evaluation | - Intervention has been evaluated using a quantitative research design for causal inference and has been found to have a statistically significant positive impact on at least one outcome of interest (see outcomes below), even if findings for other outcomes are null or negative. - Also include if quantitative research design for causal inference is part of a mixed design.   OR   - Intervention has not been evaluated but is based on a plausible logic model, programme theory, theory of change which is described in detail (not just referenced or alluded to) and some quantitative or qualitative empirical exploration of approach has been carried out (such as a pilot test or a process or formative evaluation).   OR   - Intervention has been evaluated in a previous study and found to have a statistically significant positive impact on at least one outcome of interest (see outcomes below), even if findings for other outcomes are null or negative. In this case, please take note of previous study so we can include. | - Intervention has been evaluated using any kind of quantitative research design for causal inference and has been found to have no statistically significant effect or a negative effect on any of the outcomes (see outcomes of interest below). - Intervention has been “evaluated” using a qualitative research design or based on expert opinion or satisfaction of intervention participants and is not based on a based on a plausible logic model, programme theory, or theory of change which is described in some detail. - However, evaluation of intervention based solely on expert opinion, whether or not it is based on a plausible logic model, programme theory, or theory of change will be excluded. | - Quantitative research designs for causal inference include, for instance, one group before-and-after evaluations (i.e., with no control group), pre-test/post-test with control group (whether or not randomized), interrupted time series, regression discontinuity designs, etc. |
| Outcomes of evaluation | - Reductions in the occurrence or the severity of abuse of older people. - Reduction in risk factors associated with abuse of older people (unless actual abuse is measured and findings are either null or statistically significantly negative ). - Increase in protective factors associated with abuse of older people. - Increased efforts to identify/detect, report and respond to abuse of older people. - Increased awareness and knowledge of abuse of older people. - Increased support for people who are the target of abuse of older people and for “concerned others” or perpetrators. - Reduction in psychological distress (depression, anxiety, PTSD, or other mental health measures) associated with abuse of older people. - Financial recovery, security, protection (especially for financial abuse). | - If measure of satisfaction of participants in interventions is the only outcome measured. - Opinion of experts on purported effectiveness of intervention. | - See types of abuse listed above. |
| Time period for development/ implementation/evaluation of intervention | - Any - The study/description of intervention can have been published at any time and the interventions can have been implemented at any time. No time restrictions apply. | - None |  |
| Geographical area, region, country income level in which intervention implemented. | - Any. - Countries from all regions and income levels will be included. | - None |  |
| Language of report/paper | - Any. The study/description of intervention can be in any language. No language restrictions will be applied. | - None |  |
| Additional Information: If there is not enough information in the report or paper to apply one or more criteria, then please indicate this in the appropriate column in the spreadsheet. | | | |

### Appendix 2. Criteria for Round 2

| Feasibility of Implementation | The likelihood that the prevention strategy, as designed, can be or has been implemented fully (within the organization hosting the strategy), considering the clarity of its goals, objectives, and activities; complexity and leadership requirements; financial and other costs; and training and supervision requirements. |
| --- | --- |
| Feasibility of Adoption | The extent to which a similar entity (i.e., awardee, sub-awardees, site, or organization) could adopt the prevention strategy, considering the clarity of strategy goals, objectives, and activities; complexity and leadership requirements; barriers to implementation; financial and other costs; and training and supervision requirements. |
| Transferability | The degree to which the intervention demonstrates or has the potential to be adapted for different populations or different settings. |
| Sustainability of the Strategy | The likelihood that the intervention can continue over time without special resources or extraordinary leadership. |
| Ethical Approach | The extent to which the intervention uses an ethical approach with regard to respecting individuals’ rights and protecting human dignity. |
| Reach to the Target Population | The proportion of the target/priority population that has been (or has the potential to be) reached in some way by the prevention strategy. |
| Population Plausibility | Given the nature of the prevention strategy’s activities, the likelihood that the intervention will plausibly produce the desired outcomes, leading to SV prevention. This criterion takes into consideration the theoretical underpinnings and theory of change underlying the prevention strategy. |
| Potential Impact | The extent to which the intervention has the potential for a positive impact on SV prevention outcomes and risk or protective factors. Estimate of impact is based on “face value,” conceptual logic (plausibility), and other pertinent characteristics (e.g., setting, duration, frequency, intensity) of the intervention |
| Sustainability of the Intended Outcome(s) | The likelihood the intended outcome(s) of the intervention will endure over time (e.g., seasonal strategy effect vs. long-term effect) |
| Evaluation Potential | The extent to which the intervention is ready for further evaluation (as with other criteria, responses should be based on current information available). |

### Appendix 3. Interview guide

**Abuse of Older People Intervention Accelerator** - **Interview Guide**

*This guide will have to be adapted – perhaps extensively in certain cases – for each interview depending on what information we have already gathered before the interview and what important information is still missing and we hope to collect during the course of the interview.*

***Text in italics NOT to be spoken]***

Thank you again for participating in this discussion to help us gather information on the *[name of intervention]* intervention – I very much appreciate the time you are taking to speak with me.

Before we start, we thought it would be helpful to provide a brief background on our project.

The purpose of this project is to create, within the Decade of Healthy Ageing 2021–2030, an intervention accelerator to speed up the development of effective interventions for abuse of older people in community and institutional settings within low-, middle- and high-income countries. In order to screen promising interventions that are being implemented and identify those that are ready for more rigorous testing, we have been using an adapted version of the Systematic Screening and Assessment (SSA) Method. This interview will help us to gather information on your intervention that we have been unable to find online. With this information we will be able to more thoroughly evaluate your intervention.

The interventions selected through this process will be invited to collaborate in the next phase of our project, which will involve a more rigorous and funded testing.  The interview will explore five general areas:

1. The history of the intervention;
2. The barriers faced and lessons learned from implementing this intervention;
3. Community support and partnerships necessary for the implementation of the intervention;
4. Evaluation of intervention; and
5. The funding and resources necessary for implementing the evaluation.

In addition, we will also ask you some more specific questions about the intervention to gather information we were unable to get about the intervention so far.

We value your insights across these five areas. As we have already gathered information from online sources and verified some details with you, we will prioritise exploring areas that have not been covered through public information thus far. Do you have any questions for me before we begin?

If you prefer, this interview can remain completely confidential, meaning your name will not be associated with anything you share with me today. It is your decision. Could you please indicate whether or not you prefer for it to remain confidential. Do you need a few minutes to think about it?

And so with that, I am wondering if I may have your permission to record our conversation to supplement my note-taking?

Thank you. Would you please confirm that you consent to participate in this interview, which will remain completely confidential?

____________________________________________________________________

**History**

1. Can you share with us how long this intervention has been in existence, and approximately how many years?
2. Where was the intervention originally developed? Could you provide insights into the origin of its development and the team involved?
3. We understand that effective interventions often evolve to meet changing needs. Has this intervention been adapted since its inception? If so, could you elaborate on the adaptations made and how they have contributed to its ongoing success?

**Program Design – Barriers and Lessons Learned**

1. What would you say are the main strengths of the intervention? And its main weakness?
2. List any previously faced barriers in implementing and sustaining the program?

- Political factors?
- Financial factors?
- Human resources?
- What has been done to overcome these barriers?

1. What valuable lessons have been learned from previous implementations of the intervention, and how have they informed its ongoing development and effectiveness?

**Community Support and Partnerships**

1. Have your partnered with other programs/organizations? If so, what types of benefits have you seen from partnering with them?

- How has the target population(s) benefitted from these partnerships?
- How has the community benefited from these partnerships?

1. Have these partnerships been a burden to the program or target population(s) in any way? If so, how?
2. In your opinion, who else (organizations or roles) needs to be involved with the program?

**Evaluation**

1. Can you tell us about the evaluation/s the intervention has undergone? What were the main findings (if we do not already have the findings)?
2. What were staff and stakeholders’ attitudes towards the evaluation?
3. How was it funded?
4. How were findings used? Was it considered to be useful?
5. What were some of the challenges you faced?
6. Do you know of any plans to evaluate it further?

**Funding and resources**

1. How is the program funded?

- Who funds the program and at what level?
- What other financial resources does this program receive?
- How much does the program cost to implement per participant per year?

1. Are non-monetary resources provided for the program (such as people’s time, equipment, etc.)? If so, what are they?
2. Do you know if the intervention will continue to receive resources in the future?

**Specific questions on missing information & Closing**

I have now come to the end of our questions, is there anything you would like to ask me about this study?

Thank you very much again for your time. With the information on the *[name of intervention]* we have gathered through the documentation process and this interview, we will produce a report. This report will be shared with you regardless of whether we select your intervention to be tested in the next phase of the project.

###

### Appendix 4. Report Template

- Name of intervention
- Brief description of intervention
- Brief description of any evaluation/s
- Main documents assessment based on
- Person interviewed
- Feasibility of Implementation
- The likelihood that the intervention as designed, can be or has been implemented fully (within the organization hosting the strategy), considering the clarity of its goals, objectives, and activities; complexity and leadership requirements; financial and other costs; and training and supervision requirements.
- Feasibility of Adoption
- The extent to which a similar entity (i.e., awardee, sub-awardees, site, or organization) could adopt the intervention considering the clarity of strategy goals, objectives, and activities; complexity and leadership requirements; barriers to implementation; financial and other costs; and training and supervision requirements.
- Transferability
- The degree to which the intervention demonstrates or has the potential to be adapted for different populations or different settings.
- Sustainability of the Strategy
- The likelihood that the intervention can continue over time without special resources or extraordinary leadership.
- Ethical Approach
- The extent to which the intervention uses an ethical approach with regard to respecting individuals’ rights and protecting human dignity.
- Reach to the Target Population
- The proportion of the target/priority population that has been (or has the potential to be) reached in some way by the intervention.
- Population Plausibility
- Given the nature of the intervention’s activities, the likelihood that the intervention will plausibly produce the desired outcomes, leading to SV prevention. This criterion takes into consideration the theoretical underpinnings and theory of change underlying the intervention.
- Potential Impact
- The extent to which the intervention has the potential for a positive impact on SV prevention outcomes and risk or protective factors. Estimate of impact is based on “face value,” conceptual logic (plausibility), and other pertinent characteristics (e.g., setting, duration, frequency, intensity) of the intervention
- Sustainability of the Intended Outcome(s)
- The likelihood the intended outcome(s) of the intervention will endure over time (e.g., seasonal strategy effect vs. long-term effect)
- Evaluation Potential
- The extent to which the intervention is ready for further evaluation (as with other criteria, responses should be based on current information available).
- Outcome of assessment and next steps

###

### Appendix 5. SSA Step 5 (Round 2) – Scoring System

| **No** | **Criteria** | **Poor** | **Adequate** | **Excellent** | **Insufficient Information** | **Ranking Indicators** | **Max**  **Score** |
| --- | --- | --- | --- | --- | --- | --- | --- |
| 1. | Feasibility of Implementation | The intervention cannot be fully implemented within the organization hosting the strategy or a similar entity. It lacks clear goals, objectives, and activities, and it involves high complexity and significant leadership requirements. Necessary resources are not available to support it. Additionally, financial management information is lacking, and the training and supervision requirements are unmanageable. | The intervention can be implemented in the hosting organization or a similar entity but would need some further development. | The intervention can be or has been fully implemented within the organization hosting the strategy or a similar entity. It has clear goals, objectives, and activities, with no high complexity or leadership requirements; otherwise, necessary resources are likely available to support it. If information is available, the intervention can be financially managed, and the training and supervision requirements are manageable. | Sufficient information to score this criterion is not available. | Excellent  11-15  Adequate  6-10  Very poor  0-5  Insufficient Information | 15 |
| 2. | Feasibility of Implementation in Another Country/Culture | The intervention does not have the potential to be adapted for different populations or different settings with some adaptations needed | The intervention demonstrates or has the potential to be adapted for different populations or different settings with some adaptations needed | The full intervention demonstrates or has the potential to be adapted for different populations or different settings | Sufficient information to score this criterion is not available. | Excellent  8-10  Adequate  4-7  Very poor  0-3  Insufficient Information | 10 |
| 3. | Sustainability of the Strategy | The intervention cannot continue over time without special resources or extraordinary leadership. | The intervention can continue over time with some special needs in terms of resources or leadership. | The intervention can continue over time without special resources or extraordinary leadership. | Sufficient information to score this criterion is not available. | Excellent  8-10  Adequate  4-7  Very poor  0-3  Insufficient Information | 10 |
| 4. | Ethical Approach | The intervention does not use an ethical approach with regard to respecting individuals’ rights and protecting human dignity | The intervention on the whole uses an ethical approach with regard to respecting individuals’ rights and protecting human dignity. However, there is some room for improvement. | The intervention uses an ethical approach with regard to respecting individuals’ rights and protecting human dignity | Sufficient information to score this criterion is not available. | Excellent  8-10  Adequate  4-7  Very poor  0-3  Insufficient Information | 10 |
| 5. | Potential Impact | The intervention has low potential for a positive impact on SV prevention outcomes and risk or protective factors. Estimate of impact is based on “face value,” conceptual logic (plausibility), and other pertinent characteristics (e.g., setting, duration, frequency, intensity) of the intervention | With some adaptations, the intervention has the potential for a positive impact on SV prevention outcomes and risk or protective factors. Estimate of impact is based on “face value,” conceptual logic (plausibility), and other pertinent characteristics (e.g., setting, duration, frequency, intensity) of the intervention | The intervention has the potential for a positive impact on SV prevention outcomes and risk or protective factors. Estimate of impact is based on “face value,” conceptual logic (plausibility), and other pertinent characteristics (e.g., setting, duration, frequency, intensity) of the intervention | Sufficient information to score this criterion is not available. | Excellent  14-20  Adequate  7-13  Very poor  0-6  Insufficient Information | 20 |
| 6. | Evaluation Potential | The intervention is not ready for further evaluation (as with other criteria, responses should be based on current information available). For example, outcomes cannot be quantitively measurable. It has no clear goals, objectives, and activities, with high complexity and leadership requirements. If information is available, the intervention cannot be financially managed, and the training and supervision requirements are difficult to manage. | The intervention is ready for further evaluation (as with other criteria, responses should be based on current information available), but would need some adaptation. | The intervention is ready for further evaluation (as with other criteria, responses should be based on current information available). For example, outcomes can be quantitively measurable. It has clear goals, objectives, and activities, with no high complexity or leadership requirements; otherwise, necessary resources are likely available to support it. If information is available, the intervention can be financially managed, and the training and supervision requirements are manageable. | Sufficient information to score this criterion is not available. | Excellent  11-15  Adequate  6-10  Very poor  0-5  Insufficient Information | 15 |
| 7. | Availability and Willingness of partners to Collaborate in Future Evaluations | N/A | N/A | N/A | N/A | N/A | N/A |
| 8. | Availability of Materials | No intervention materials available. | Some intervention materials are available, but others are missing. Or materials are available but would need to be updated or adapted. | Intervention materials are available. | Sufficient information to score this criterion is not available. | Excellent  8-10  Adequate  4-7  Very poor  0-3  Insufficient Information | 10 |
| 9. | Resources Required | Acquiring the necessary resources (e.g., staff, venue, etc.) for implementing the intervention is either impossible or extremely difficult. | The necessary resources (e.g., staff, venue, etc.) for implementing the intervention can be acquired with a reasonable amount of effort. | The necessary resources (e.g., staff, venue, etc.) for implementing the intervention, or most of them, are either already available or can be acquired with relative ease. | Sufficient information to score this criterion is not available. | Excellent  8-10  Adequate  4-7  Very poor  0-3  Insufficient Information | 10 |

Note: N/A = not scored.
